# Which factors determine the patients’ care intensity for surgeons and surgical nurses? A conjoint analysis

**DOI:** 10.1186/1472-6963-14-S2-P135

**Published:** 2014-07-07

**Authors:** Catharina J van Oostveen, Hester Vermeulen, Els J Nieveen van Dijkum, Dirk J Gouma, Dirk T Ubbink

**Affiliations:** 1 Department of Quality Assurance & Process Innovation, Academic Medical Center, Amsterdam, The Netherlands; 2 Department of Surgery, Academic Medical Center, Amsterdam, The Netherlands; 3 Amsterdam School of Health Professions, University of Amsterdam, Amsterdam, The Netherlands

## Background

In the Netherlands more than 50% of the adverse events are related to surgical procedures. Therefore the risks of direct harm and high hospitalization costs are substantial. This makes adequate staffing of surgeons and nurses an important issue on clinical wards as insufficient staffing is related to higher mortality and morbidity rates. Clinical surgeons and nurses sometimes perceive a high workload on their surgical wards, which may influence admission decisions and staffing policy. Yet, it is unclear what the relative contribution is of various patient and care characteristics to the perceived patients’ care intensity and whether differences exist in the perception of surgeons and nurses.

## Materials and methods

Dutch surgeons and surgical nurses were invited by means of internet and e-mail calls to rate 20 virtual clinical scenarios regarding patient care intensity on a 10-point Likert scale. The scenarios described patients with 5 different surgical conditions: cholelithiasis, a colon tumour, a pancreas tumour, critical leg ischemia, and an unstable vertebral fracture. Each scenario presented a mix of 13 different attributes that possibly influence care intensity, as derived from a systematic literature review. These attributes referred to the patients’ condition, physical symptoms, and admission and discharge circumstances.

## Results

A total of 82 surgeons and 146 surgical nurses completed the questionnaire, resulting in 4560 rated scenarios and 912 per condition. For surgeons, 6 out of the 13 attributes contributed significantly to care intensity: age, polypharmacy, medical diagnosis, complication level, ICU-stay, and ASA-classification. Conversely, multidisciplinary care did not contribute significantly. For nurses, the same six attributes contributed significantly, but also BMI, nutrition status, admission type, patient dependency, anxiety or delirium during hospitalization, and discharge type. Both professionals ranked ‘complication level’ as having the highest impact.

## Conclusions

Surgeons and nurses differ in their perception of patient caring intensity. Awareness of these factors may help managers optimise the work processes on clinical wards, in terms of staff planning and aligning the activities of surgeons and nurses. Furthermore, a shared workload language would avail surgeons and nurses in understanding, appreciating and respecting each other’s work. This may eventually have a positive impact on patient safety.

